# Data of RNA-seq transcriptomes of gastrocnemius muscle, epididymal adipose tissue in obese rats under normoxia/hypoxic exercise environments

**DOI:** 10.1016/j.dib.2024.110134

**Published:** 2024-02-01

**Authors:** Wen Ma, Youhan Liu, Chang Meng, Ying Luo, Qinglu Wang

**Affiliations:** a College of Sport and Health, Shandong Sport University, Jinan, Shandong 250102, China; b Key Laboratory of Biomedical Engineering & Technology of Shandong High School, Qilu Medical University, Zibo 255213, China; c Department of Clinical laboratory, Zibo Central Hospital, Zibo 255000, China

**Keywords:** Obesity rats, Hypoxia exercise, Normoxia exercise, Adipose tissue, Muscle tissue

## Abstract

Studies on the crosstalk between muscle and adipose tissue can provide beneficial help in elucidating the pathogenesis and treatment of obesity-related diseases [1]. In this data article, we performed RNA sequence analysis of mRNA isolated from epididymal adipose tissue and gastrocnemius muscle tissue in obese rats. Twenty-two samples were selected for gene expression analysis. Raw data from the Illumina Hiseq™ platform sequencer was used for differential gene expression analysis using DESeq and deposited in the GEO public repository under accession number GSE237950. With the economic development and the change of people's lifestyle, obesity has become a major public health problem that endangers global health. Obesity is a metabolic disorder caused by excessive accumulation of white adipose tissue, which can further induce metabolic syndrome such as insulin resistance, type 2 diabetes, and cardiovascular and cerebrovascular diseases. Studies have shown that altitude hypoxic exercise can not only improve muscle buffering capacity and body performance, but also reduce body weight and body fat more significantly. In many countries, it has been used as a treatment program for obesity diseases [2]. Hypoxic exercise can improve lipid metabolism, reduce blood lipid levels, inhibit fatty acid synthesis, and promote fatty acid decomposition and oxidation, which is the mechanism of hypoxic exercise to significantly reduce weight and fat. However, the mechanism of the cross-talk between muscle and fat tissue is not well understood under hypoxia exercise and normoxia exercise conditions. The data contained rat's four different states: normoxia quiet, normoxia exercise, hypoxic quiet, and hypoxic exercise. RNA-seq data will provide insights into the cross-talk between muscle and fat, and the mechanisms of fat metabolism. The data of this study have not been published and are hereby published on this platform to study the cross talk between muscle tissue and adipose tissue in rats under different oxygen content and exercise environment.

Specifications Table

**Table:** 

Subject	Biological and Molecular Sciences, Exercise physiology
Specific subject area	Exercise and lipid metabolism, Muscle and adipose tissue cross-talk
Data format	Raw and analyzed, Filtered raw reads (FASTQ)
Type of data	Tables, Chart, Graph, Figures, RNA-seq raw data
Data collection	Total RNA was extracted from fat normoxia exercise group (FCE, *n* = 3), fat normoxia quiet group (FC, *n* = 3), fat hypoxic quiet group (FH, *n* = 2) and fat hypoxia exercise group (FHE, *n* = 2), muscle normoxia exercise group (MCE, *n* = 3), muscle normoxia quiet group (MC, *n* = 3), muscle hypoxic quiet group (MH, *n* = 3), and muscle hypoxia exercise group (MHE, *n* = 3)
Data source location	Shandong Sport University, Jinan, Shandong, China
Data accessibility	Repository names: Gene Expression Omnibus Data identification number: GSE237950 Direct URL to data: https://www.ncbi.nlm.nih.gov/geo/query/acc.cgi?acc=GSE237950

## 1 Value of the Data

• The cross-talk study of gastrocnemius muscle and epididymal adipose can provide mechanisms of action between tissues in glucose metabolism, lipid metabolism, extracellular vesicles, and cytokine responses.
• Which method increases the catabolism of adipose tissue between hypoxia and normoxia exercise.
• The data can be used to analyze gene expression and signaling pathway changes in gastrocnemius tissue and epididymal adipose tissue under normoxic quiescence, hypoxic quiescence, normoxic exercise and hypoxic exercise conditions.

## 2 Objective

The Objective of this study was to analyze the cross talk between muscle tissue and adipose tissue in rats under different oxygen content and exercise environment, to provide help for relevant researchers.

## 3 Data Description

Gastrocnemius muscle and epididymal adipose for RNA-seq were isolated from obesity rat model: Total RNA was extracted from epididymal adipose tissue and gastrocnemius muscle tissue. Samples for sequencing analysis were grouped as fat normoxia exercise group (FCE, *n* = 3), fat normoxia quiet group (FC, *n* = 3), fat hypoxic quiet group (FH, *n* = 2) and fat hypoxia exercise group (FHE, *n* = 2), muscle normoxia exercise group (MCE, *n* = 3), muscle normoxia quiet group (MC, *n* = 3), muscle hypoxic quiet group (MH, *n* = 3), and muscle hypoxia exercise group (MHE, *n* = 3). Raw data from the Illumina Hiseq™ platform sequencer is stored as FASTQ and gene abundance data in the Gene Expression Omnibus (GEO) (accession number: GSE237950) [3]. The accession number for individual samples in GEO database in Table 1. Thereafter, libraries were prepared and checked for quality using the Bioanalyzer 2100. The raw RNA-seq reads were obtained with the Illumina Hiseq™ platform [4]. The results are stored in the FASTQ (fq) file format containing sequence information of sequencing sequences (reads) and their corresponding sequencing quality information. Then, we carried out different bioinformatic analyses of the raw RNA-seq data including: Quality assessment of the sequencing data, Mapping Results, Redundant sequence analysis, Single Nucleotide Polymorphisms analysis, Variable shear analysis, the gene expression levels analysis, Co-expression of Wayne diagrams, Comparative analysis of the expression levels, Differential expression analysis, Functional annotation of the differential genes, Differential gene KEGG pathway diagram, Functional enrichment analysis of the differential genes, Functional enrichment for the association analysis and so on [5].

**Table 1:** List of accession number of epididymal adipose tissue and gastrocnemius muscle tissue in obese rats’ transcriptome in GEO database.

Sample	Type	Treatment	Geo accession number
FCE1	Adipose tissue	Normoxia exercise	GSM7656816
FCE2	Adipose tissue	Normoxia exercise	GSM7656817
FCE3	Adipose tissue	Normoxia exercise	GSM7656818
FHE2	Adipose tissue	Hypoxia exercise	GSM7656819
FHE3	Adipose tissue	Hypoxia exercise	GSM7656820
FC1	Adipose tissue	Normoxia quiet	GSM7656821
FC2	Adipose tissue	Normoxia quiet	GSM7656822
FC3	Adipose tissue	Normoxia quiet	GSM7656823
FH1	Adipose tissue	Hypoxic quiet	GSM7656824
FH3	Adipose tissue	Hypoxic quiet	GSM7656825
MCE1	Musculature	Normoxia exercise	GSM7656826
MCE2	Musculature	Normoxia exercise	GSM7656827
MCE3	Musculature	normoxia exercise	GSM7656828
MHE1	Musculature	Hypoxia exercise	GSM7656829
MHE2	Musculature	Hypoxia exercise	GSM7656830
MHE3	Musculature	Hypoxia exercise	GSM7656831
MC1	Musculature	Normoxia quiet	GSM7656832
MC2	Musculature	Normoxia quiet	GSM7656833
MC3	Musculature	Normoxia quiet	GSM7656834
MH1	Musculature	Hypoxic quiet	GSM7656835
MH2	Musculature	Hypoxic quiet	GSM7656836
MH3	Musculature	Hypoxic quiet	GSM7656837

**Fig. 1:**
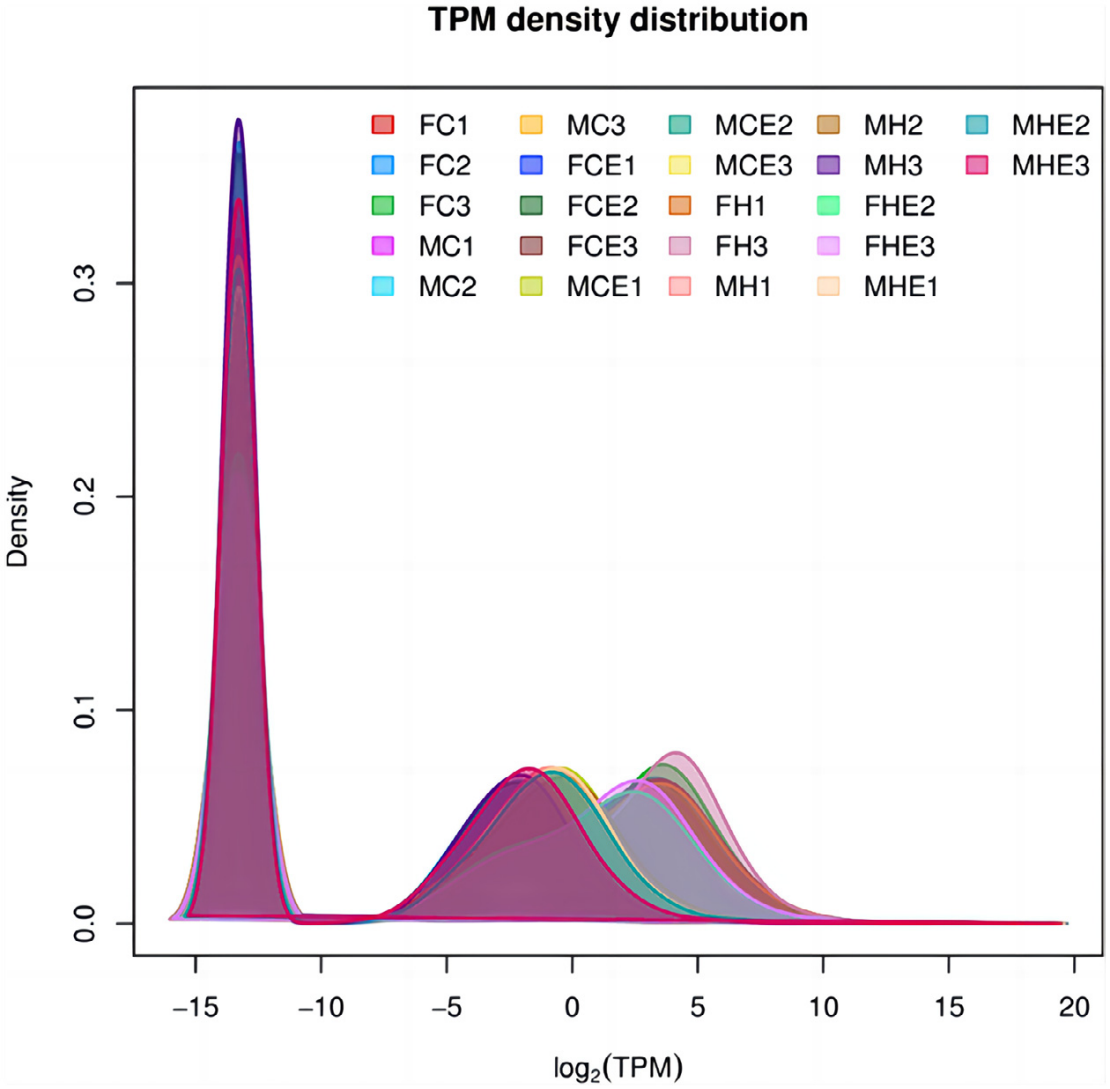
The density plot of the gene expression levels. The horizontal axis is the log (TPM) value, the higher the gene expression; the vertical axis is the corresponding relative density value, which is the number of genes expressed in the horizontal axis / the total number of the genes expressed. Each color in the figure represents a sample, the area of each region is 1, and the peak of the density curve indicates the highest concentration of gene.

**Fig. 2:**
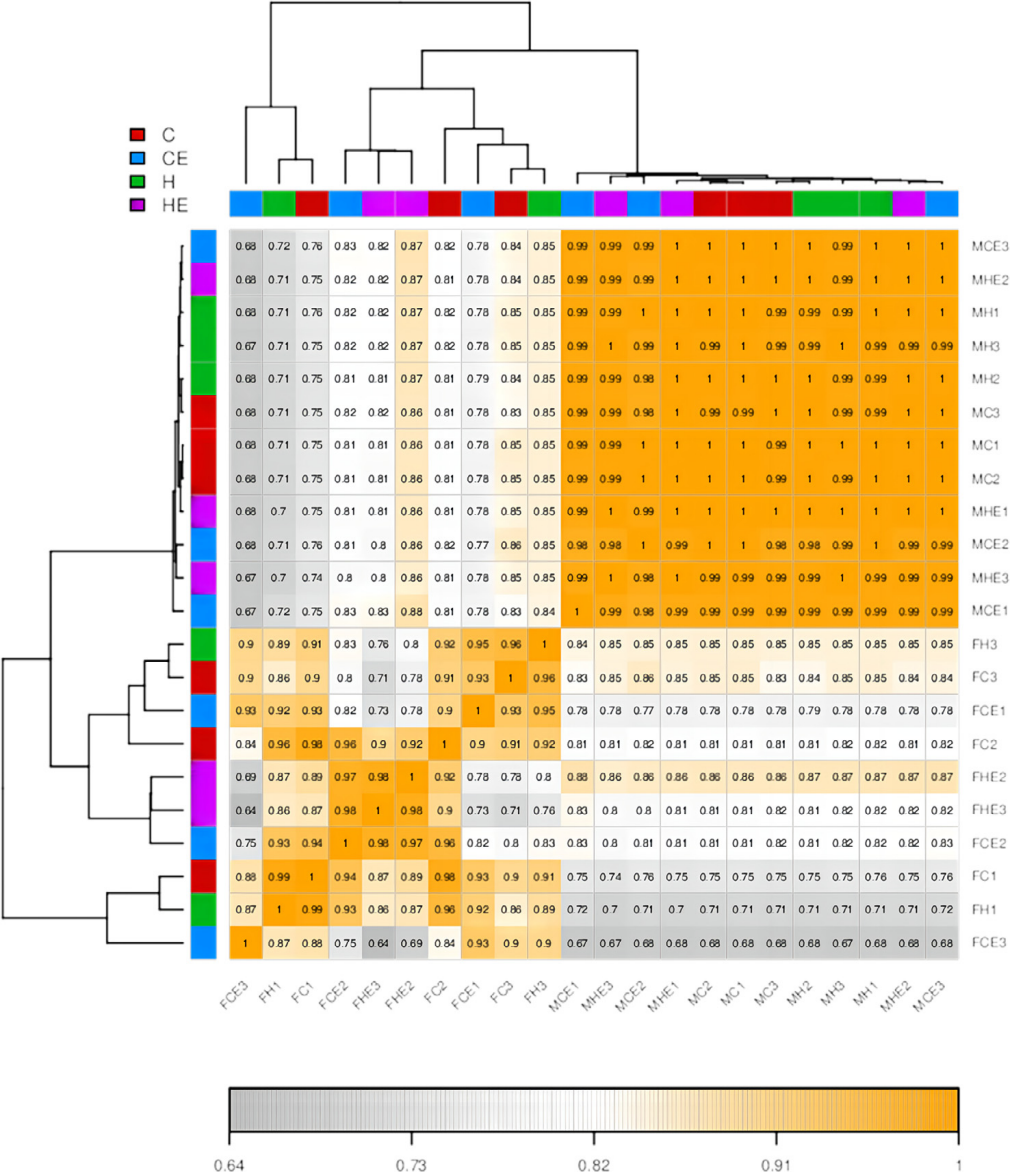
Heat map of correlation analysis between samples.

In RNA-seq analysis, we can estimate the expression level of genes by counting sequencing sequences (reads) mapping to the genomic regions or exonic regions of the gene. Fig. 1 represents the gene expression level results for 22 samples. The correlation of gene expression level is an important indicator to describe whether the data selection is reasonable. The closer the correlation coefficient between samples is to 1, the higher the similarity of gene expression pattern between sample, to explore the cross talk between muscle and adipose tissue. Fig. 2 shows the correlation between samples, the color block represents the correlation index value, the gray the color indicates the correlation index between samples, the yellow the color is the correlation index is higher. C, CE, H and HE represent four different intervention methods, C represents normoxia quiet, CE represents normoxia exercise, H represents hypoxic quiet, and HE represents hypoxia exercise. The analysis showed that there was a strong correlation between adipose tissue and muscle tissue in the four different groups under HE conditions. For example, the expression of FHE2 was highly correlated between muscle tissue and adipose tissue under hypoxic. This study further demonstrated that hypoxic exercise enhanced the crosstalk between adipose tissue and muscle tissue.

## 4 Experimental Design, Materials and Methods

### 4.1 Experimental design

All animal experiments were approved by the Animal Ethics Committee of Shandong Sport University (No. 2015206). Seventy healthy 3-week-old male SD rats weighing 57 ± 5 g were purchased from Wuhan Hualian Science Biotechnology Co., Ltd. Ten rats were randomly selected as the general feed group, and the remaining rats were fed high-fat feed to construct an obese rat model. These rats are maintained in a facility with a temperature of 23 ± 2 °C and a humidity of 55 ± 2% for 12 h in a light/dark cycle. The rats in the control group were fed regular feed, and the obese rat model group was fed 60% high-fat feed. These mice are weighed at a fixed time each week. After 8 weeks, obese rats were tested for successful modeling. The success of the modeling was that its weight exceeded 20% of the average weight of the control group. Male rat serum total cholesterol (TC), triglycerides (TG), low-density lipoprotein (LDL-C), and high-density lipoprotein (HDL-C) were measured weekly by Elisa as standard. If the serum TG, TC and LDL-C were increased significantly *(P < 0.05)* and HDL-C was significantly decreased *(P < 0.05)*, the modeling was considered successful. Forty rats were randomly divided into 4 groups: normoxia quiet group (C), normoxia exercise group (CE), hypoxic quiet group (H) and hypoxic exercise group (HE). In this study, a total of 40 rats were selected for modeling, and 24 rats met the modeling criteria, of which two rats were excluded because of unqualified adipose tissue RNA quality inspection, and finally 22 rats were selected for RNA-seq analysis. The 22 rats included fat normoxia exercise group (FCE, *n* = 3), fat normoxia quiet group (FC, *n* = 3), fat hypoxic quiet group (FH, *n* = 2) and fat hypoxia exercise group (FHE, *n* = 2), muscle normoxia exercise group (MCE, *n* = 3), muscle normoxia quiet group (MC, *n* = 3), muscle hypoxic quiet group (MH, *n* = 3), and muscle hypoxia exercise group (MHE, *n* = 3).

**Fig. 3:**
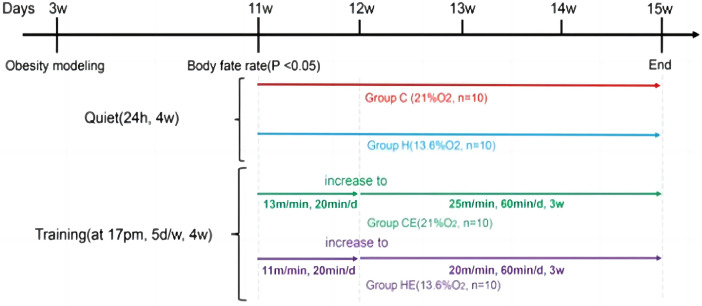
Schematic diagram of animal experiment process.

Rats in the quiet group did not undergo exercise intervention and kept the cage active for four weeks for 24 h per day. Oxygen concentration was 21% in Group C and 13.6% in Group H. Rats in the exercise group underwent treadmill training, and training started at 17:00 daily, 1 h/d, 5d/w. Week 1 is the adaptive training: The treadmill speed increased from 13m/min to 25 m/min, from 11 m/min to 20 m/min, and the duration increased from 20 min/d to 60 min/d in both CE and HE groups, and the speed and duration remained unchanged for the next 3 weeks(Fig. 3).

Laboratory animals were banned from a drinking diet 12 h prior to sampling. Peritoneal anesthesia was performed with 10% hydrated trichloroacetaldehyde (dose calculated at 4 ml/kg body weight) and then the rat was killed and isolated epididymal fat and gastrocnemius muscle. Epididymal fat and gastrocnemius muscle placed into liquid nitrogen and sent to Biotechnology (Shanghai) Co., Ltd. for tissue RNA-seq analysis.

### 4.2 RNA-seq and data quality control analyses

The Illumina Truseq mRNA stranded RNA-Seq Library Prep Kit protocol was followed. The data was checked for quality and quantified using the Bioanalyzer 2100 at Sangon Bioengineering Co., LTD (shanghai). The quality of the sequencing output was assessed using FastQC [6]. Information such as raw data quality values were counted, and the sequencing data quality of the samples was visually assessed using FastQC. Quality filtering and removal of residual adaptor sequences was conducted on read pairs using Fast QC. Ten thousand sequences were randomly selected from the clean data for blastn alignment with the NCBI NT database, evalue <=1e−10 and similarity. When the alignment results were >90% and coverage >80%, the species distribution was calculated and the contamination was detected [7].

For RNA-seq analysis, rat genome of reference: ftp://ftp.ensembl.org/pub/release-99/fasta/rattus_norvegicus/dna/.

Rat's annotation files: ftp://ftp.ensembl.org/pub/release-99/gtf/rattus_norvegicus.

**Fig. 4:**
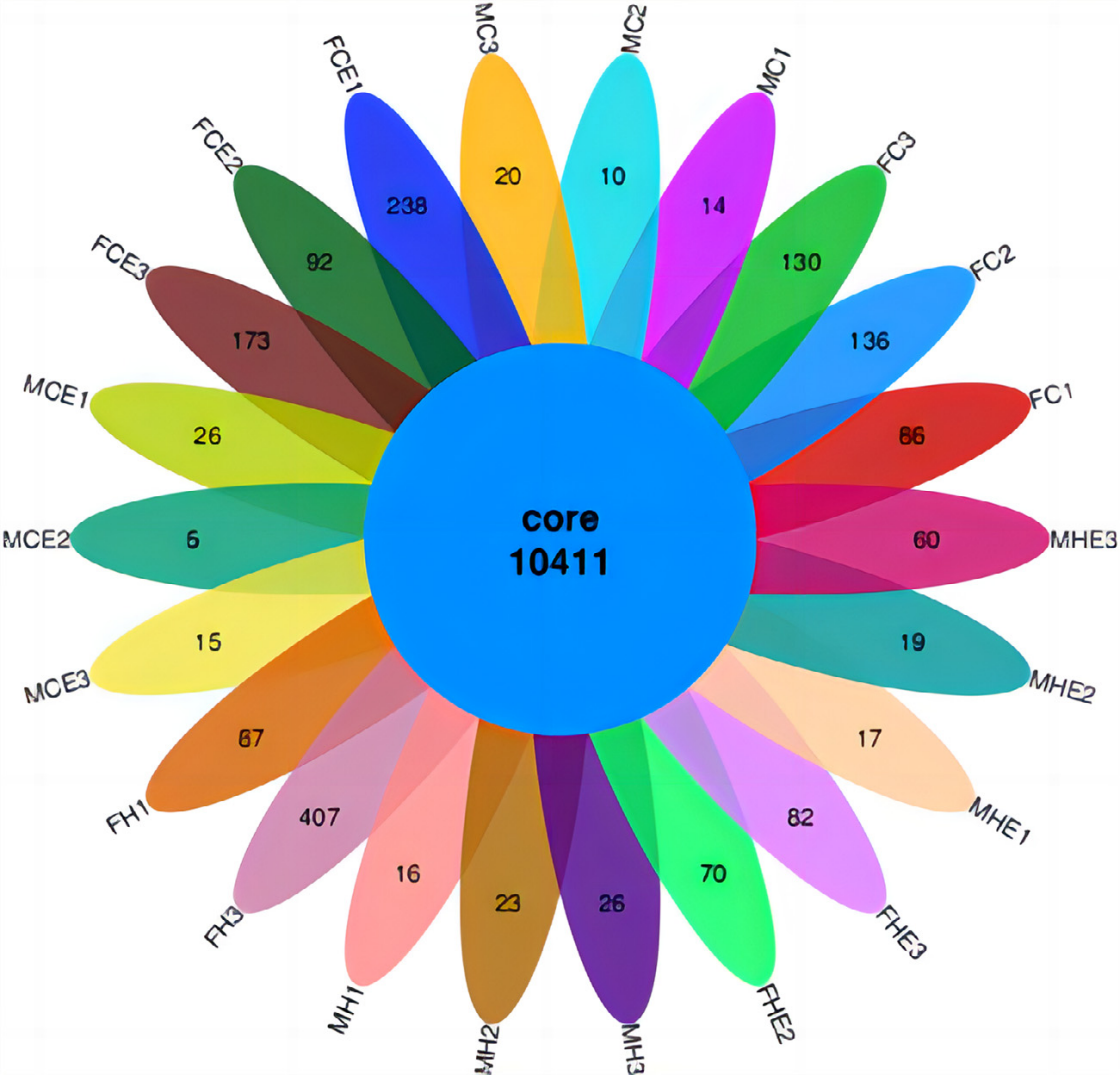
Co-expression of Wayne diagrams.

The expression level of a gene can be estimated by the number of sequencing sequences (reads) that map to a genomic region or a gene's exon region. HISAT2 was used to align the effective sample data to the reference genome, and BED Tools was used to analyze the statistical analysis of gene coverage and the distribution of sequencing sequences on chromosomes. The reference genome was used as the reference sequence, and the sequencing sequences after quality control were aligned to the reference genome using HISAT2 and statistically aligned by RSeQC. The VENN graph (Fig. 4) can be used to count the number of some and unique expressed genes (TPM> 0) in 22 samples, visually showing the similarity and overlap of the number of expressed genes in the sample. Qualimap software was used to calculate the proportion of reads in each genome structure, including Exon, Intron, and Intergenic region, after reads alignment to the reference genome. Table 2 shows the proportion of genomic reads accounted for by exons, introns, and intergenic regions and the total number of reads, the number of reads mapped to exons, introns, and the number of unaligned reads for the 22 samples.

**Table 2:** The proportion of genomic reads accounted for by exons, introns, and intergenic regions and the total number of reads, the number of reads mapped to exons, introns, and the number of unaligned reads for the 22 samples.

Sample	Exon		Intron		Intergenic			Total reads
	Percentage	number of reads	Percentage	number of reads	Percentage	number of reads	Number of unaligned reads	
FC1	82.86 %	38,387,936	6.02 %	2,789,431	11.12 %	5,150,278	472,693	46,800,338
FC2	82.39 %	41,219,696	6.49 %	3,246,852	11.12 %	5,562,558	414,951	50,444,057
FC3	78.92 %	29,870,581	8.93 %	3,380,459	12.14 %	4,595,834	286,470	38,133,344
FCE1	82.35 %	36,893,388	5.57 %	2,496,055	12.07 %	5,409,058	463,559	45,262,060
FCE2	82.57 %	40,706,837	5.90 %	2,908,628	11.53 %	5,686,421	372,608	49,674,494
FCE3	78.53 %	35,977,838	7.44 %	3,408,696	14.03 %	6,428,692	409,361	46,224,587
FHE2	82.70 %	34,891,605	4.55 %	1,921,236	12.75 %	5,377,187	289,873	42,479,901
FHE3	81.89 %	41,853,747	4.97 %	2,539,532	13.14 %	6,717,255	272,268	51,382,802
FH1	82.88 %	35,959,158	6.17 %	2,675,146	10.95 %	4,751,309	472,724	43,858,337
FH3	72.47 %	34,035,867	13.32 %	6,254,670	14.21 %	6,673,885	371,209	47,335,631
MCE1	89.36 %	41,541,193	0.88 %	409,008	9.76 %	4,535,798	44,217	46,530,216
MCE2	90.55 %	45,870,593	0.32 %	161,739	9.13 %	4,624,021	20,052	50,676,405
MCE3	90.25 %	43,967,634	0.77 %	373,710	8.99 %	4,378,674	37,559	48,757,577
MHE1	90.22 %	35,457,888	0.94 %	367,678	8.84 %	3,474,462	37,408	39,337,436
MHE2	90.23 %	38,387,390	0.92 %	393,144	8.85 %	3,763,967	36,577	42,581,078
MHE3	89.30 %	43,633,447	0.55 %	267,596	10.15 %	4,961,511	17,273	48,879,827
MC1	91.15 %	28,971,761	0.58 %	185,073	8.27 %	2,627,563	22,020	31,806,417
MC2	91.13 %	36,342,868	0.28 %	110,854	8.59 %	3,426,651	15,153	39,895,526
MC3	89.71 %	47,218,461	0.89 %	470,105	9.40 %	4,946,785	64,918	52,700,269
MH1	90.44 %	35,665,781	0.72 %	282,537	8.85 %	3,489,698	29,739	39,467,755
MH2	89.29 %	44,223,662	1.56 %	771,514	9.16 %	4,535,136	58,509	49,588,821
MH3	89.60 %	34,337,761	0.31 %	120,688	10.08 %	3,863,532	8736	38,330,717

## Limitations

Not applicable

## Ethics Statement

The animals we selected were male SD rats, all experiments were conducted in accordance with the National Institutes of Health Guidelines for the Care and Use of Experimental Animals (NIH Publication No. 8023, revised 1978). Approval for this study was provided by the Shandong Sport University Animal Ethics Committee (China).

## CRediT Author Statement

Conception and design of the work: Wen Ma and Chang Meng; acquisition and analysis of data: Wen Ma and Youhan Liu; interpretation of data: Wen Ma and Chang Meng; writing and preparing the original draft: Wen Ma and Ying Luo; writing, reviewing, and editing the paper: Qinglu Wang and Ying Luo.

## Data Availability

Data of RNA-seq transcriptomes of gastrocnemius muscle and epididymal adipose tissue in obese rats changes in hypoxic exercise environments (Original data) (GEO)
